# An Illusory Motion in Stationary Stimuli Alters Their Perceived Duration

**DOI:** 10.3390/vision7030061

**Published:** 2023-09-18

**Authors:** Giulio Contemori, Giulia Meneghini, Luca Battaglini

**Affiliations:** 1 Department of General Psychology, University of Padova, Via Venezia 8, 35131 Padova, Italy; luca.battaglini@unipd.it; 2 Padova Neuroscience Center, University of Padova, 35131 Padova, Italy; giulia.meneghini.2@phd.unipd.it; 3 Department of Neuroscience, University of Padova, 35131 Padova, Italy

**Keywords:** illusion, vision, time, perception, discrimination, movement

## Abstract

Despite having equal duration, stimuli in physical motion are perceived to last longer than static ones. Here, we investigate whether illusory motion stimuli produce a time-dilation effect similar to physical motion. Participants performed a duration discrimination task that compared the perceived duration of static stimuli with and without illusory motion to a reference stimulus. In the first experiment, we observed a 4% increase in the number of “longer” responses for the illusory motion images than static stimuli with equal duration. The time-dilation effect, quantified as a shift in the Point of Subjective Equality (PSE), was approximately 55 ms for a 2-second stimulus. Although small, the effect was replicated in a second experiment in which the total number of standard-duration repetitions was reduced from 73 to 19. In the third experiment, we found a positive linear trend between the strength of the illusory motion and the magnitude of the time-dilation effect. These results demonstrate that, similar to physical motion stimuli, illusory motion stimuli are perceived to last longer than static stimuli. Furthermore, the strength of the illusion influences the extent of the lengthening of perceived duration.

## 1. Introduction

Perception is influenced both by the accessibility to environmental information and by the functional architecture of our perceptual system. One of the tasks of our perceptual system is to strengthen and interpolate sensory input so that we can interpret the surrounding scene even in the event of degraded or missing information. However, during this process, the direct link to physical reality can be lost and some perceptual discrepancies can be introduced [1]. In most cases, these discrepancies go unnoticed, but occasionally they produce illusions that dramatically alter our judgments. In this context, a visual illusion can be seen as the failure of a perceptual heuristic that is adaptive in other instances [2,3]. Under unfavorable conditions, all perceptual domains fall prey to illusions, and time is no exception [4].

Chronoception plays a crucial role in our ability to carry out daily activities, and numerous studies have been conducted to investigate biases in this domain. Perceptual distortions in chronoception can affect judgments of order, simultaneity, and duration [5,6]. The influence of different contextual properties, intrinsic or extrinsic to the stimulus, has been explored, revealing discrepancies between physical and perceptual time (for a review, see Gorea, 2011 [7]). In 1995, Brown demonstrated that a moving object is perceived to last longer than a static one, with faster speeds inducing a greater lengthening of perceived time [4]. Subsequent studies have consistently replicated this finding and identified various characteristics of moving stimuli that contribute to this time-dilation effect. It is now understood that temporal dilation increases with higher temporal frequencies [8], higher speeds [9], looming motion [10], implied motion [11], stimulus deceleration [12] and increased motion coherence [13].

It is unclear whether there is an amodal central mechanism for time perception [14,15]. The most widely accepted model of interval timing is the Pacemaker-Accumulator (PA) model, introduced by Treisman in 1963 [16]. This model consists of two hypothetical components: a pacemaker-accumulator unit responsible for generating and counting pulses and a reference memory unit that encodes the total number of pulses representing the timed interval. Expanding on this model, Gibbon et al. (1984) incorporated a decision component that compares the pulse count in the accumulator with a reference memory, allowing temporal judgments [17].

One way to interpret motion-induced time dilation is to consider that higher speed stimuli can potentially accelerate the pacemaker, leading to a longer perceived duration [9,18]. Importantly, this interpretation remains valid even when considering other interval timing models, such as the Beat Frequency model or the Change model [4]. However, since the duration dilation effects do not transfer between different modalities [19], Kaneko and Murakami (2009) propose a revised PA model that does not require a general pacemaker but assumes the presence of an internal clock specific to the visual modality [9]. Based on this model, the expectation is to replicate the time-dilation effect using stationary stimuli, provided that they have the potential to accelerate the pacemaker’s rhythm.

In this study, we investigate whether illusory motion stimuli generate motion-induced duration dilation in a manner similar to physical motion stimuli. Illusory motion stimuli are a particular class of static images with characteristics that evoke a clear sensation of motion in the viewer. A famous example is the so-called “rotating snakes”, a spatio-temporal illusion in which both spatial layout and temporal stimulation contribute to inducing a strong motion perception that is consistent among viewers [20]. In such images, spatial transitions in local contrast provide the spatial component, while small transient oculomotor events such as microsaccades contribute to the temporal component [21,22].

Within the psychophysical literature, it has been suggested that human visual processing comprises two main temporal channels: a sustained channel and a transient channel, which roughly correspond to the parvocellular and magnocellular systems, respectively [23,24]. Perception of this illusion strongly involves the transient system, which favors low spatial and high temporal frequencies [25]. Furthermore, functional magnetic resonance studies have revealed the activity of the cortical network for the processing of motion from V1 to MT+ in response to this illusion [26,27,28].

Notably, illusory motion and physical motion not only share neural underpinnings but also exhibit behavioral similarities in various situations. For instance, in a series of four experiments, standard visual search was used to explore whether the onset of illusory motion pre-attentively guides vision in the same way that the onset of real motion is known to do. The results showed that the illusory motion “pops out” the same way as the physical motion, irrespective of the size of the set of elements [29]. Another study meticulously examined the impact of real, illusory, and implied motion on pupil diameter. The findings illustrated that both illusory and physical motion induces greater pupillary dilation compared to control photos representing static subjects, with physical motion inducing greater dilation [30]. Furthermore, for both illusory [30,31,32] and physical motion [33], as motion speed escalates, pupil dilation gradually increases. Another study provided evidence that illusory motion evokes self-motion, known as vection, similar to physical display motion [34]. In summary, both illusory and physical motion activate analogous neural networks and elicit comparable attentional and physiological responses. Given these findings, we hypothesized that illusory motion would have a similar effect to physical motion on the pacemaker’s rhythm and, consequently, extend the perceived duration of events.

To examine whether illusory motion influences perceived duration, we designed a series of experiments using a time discrimination task, in which the duration of images with and without illusory motion was compared to a reference duration. In Experiment 1, our aim was to determine whether illusory motion images were perceived to last longer compared to static images. In Experiment 2, we investigated whether cognitive factors, such as memory load, interacted with the expansion of perceived duration. Lastly, in Experiment 3, we aimed to establish whether the perceived duration varied with the perceived strength of the illusion, drawing a parallel with findings on the speed of physical motion [9].

## 2. Experiment 1

In this experiment, using a temporal discrimination task, we evaluated the difference in perceived duration between static and illusory motion images. Participants were asked to compare the duration of a static or illusory moving visual stimulus with a previously memorized reference duration. We hypothesized that illusory stimuli would elicit a higher number of “longer” responses compared to static stimuli, indicating temporal dilation. To test this hypothesis, we employed an information-based model selection and an omnibus test. Furthermore, we compared the Point of Subjective Equality (PSE) derived from psychometric functions for the two types of stimuli. The PSE represents the duration of the target at which subjective perception equals the previously memorized reference duration. To assess whether the illusory image was effective in eliciting motion sensation, at the end of the experiment participants were also asked to rate the perceived strength of the illusion.

### 2.1. Materials and Methods

**Figure 1:**
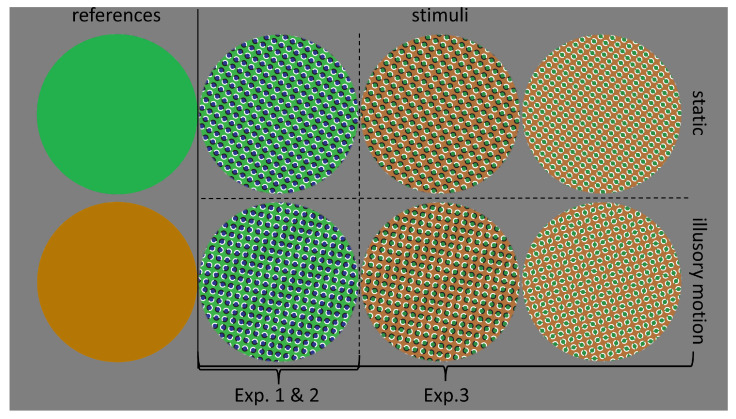
Stimuli employed in this study. The leftmost column comprises the reference stimuli used to establish the standard duration. The subsequent columns contain the target stimuli. In the lower row, we present the illusory stimuli. Specifically, the second left column contains stimuli that induced a strong illusion, followed by stimuli that elicited a mild illusion in the next column, and stimuli that produced a weak illusion in the rightmost column. In the upper row, we display their static counterparts. In Experiments 1 and 2, we utilized the green-blue texture pair along with the solid green reference stimuli. Additionally, in Experiment 3, we introduced the green-orange pairs and the solid orange reference stimuli.

The target stimuli were obtained from existing peripheral drift images [31] and consisted of two sets of texture coupled in pairs of repeating asymmetric patterns (RAPs) (see Figure 1).

The images in each couple were characterized by an equal global pattern but different physical orientation of local elements. The images were static, meaning there were no changes in luminance over time. However, one image in each pair elicited a motion illusion while the other did not. The stimulus diameter was 25 degrees of visual angle and occupied nearly the entire vertical axis of the screen, leaving approximately a 1 cm margin both at the top and bottom. In addition, a black fixation dot with a diameter of 0.3 degrees of visual angle appeared at the center of the screen for the duration of the stimulus.

The illusory images had three key characteristics that generated a strong sensation of motion: (1) the presence of a repeated asymmetrical pattern (RAP) [35], (2) alternating high and low luminance regions [36,37], and (3) alternating opposite colors [38]. RAPs allow the control of the perception of illusory motion by manipulating the physical orientation of local elements within the global pattern.

Illusory images in this study elicit an undulatory motion perception rather than a directional motion perception. Given the inherent difficulty in quantifying the “speed” of this type of motion, to assess the magnitude of the illusory motion sensation, a subjective measure was employed. Previous research by Backus and Oruç (2005) [36] with similar RAP stimuli showed that duration affects the perceived “speed” of the illusion when contrast and luminance are matched. They found that the perceived speed initially starts high, decreases, and stabilizes after about 2.5 s. In subsequent seconds, the perceived speed remains relatively stable. In this study, we employed suprasecond duration levels, which were more likely to ensure a stable perception of speed over time.

In our first experiment, we used a light-green and blue texture along with a solid green reference image (Figure 1). We presented this pair with 11 different duration levels (ranging from 1.25 to 2.75 s), while the reference duration was 2.0 s. The experimental session consisted of 220 trials, divided into 22 blocks of 10 trials each. The session lasted approximately 30 min. At the beginning of the session, participants received on-screen instructions and were asked to read and follow them carefully. The task began with the presentation of the reference image ten times. Participants were instructed to memorize its duration. The reference duration was also presented three times at the beginning of each subsequent block, totaling 73 repetitions. In experimental trials, the participants had to compare the duration of the target stimulus with the reference stimulus. They were instructed to press the “Z” key if the target appeared shorter than the reference and the “M” key if it appeared longer. A keyboard with a qwerty-type layout was used. The targets were two green/blue textures with symmetric (static) or asymmetric (illusory) patterns (Figure 1). Each target was randomly presented 10 times per duration (10 repetitions × 11 duration levels). Although imposing restrictions on participant eye movements could potentially diminish the illusion effect [22,39], we opted to introduce a fixation point. This choice was made to enhance the reproducibility of our findings while mitigating potential confounding factors. A black dot measuring 0.3 degrees in diameter was displayed in the center of the screen throughout the stimulus presentation. Participants received instructions to look at the center of the stimulus and to avoid blinking during its on-screen duration. The task was self-paced, and participants were asked to blink only between trials.

The order of stimulus presentation was randomized for each participant, and no feedback was provided during the experiment. There was no specified maximum response time between trials or blocks, allowing participants to take their time to complete the task. However, they were instructed to avoid prolonged and frequent pauses to remain engaged in the task. Participants were explicitly instructed not to count or use any other artificial means to measure duration but to rely on their subjective feeling of time.

At the end of the task, we presented one static image and one illusory image separately to the participants, without a time limit. We asked them to rate the intensity of motion experience elicited by each stimulus on a scale from zero to nine with zero being “no motion” and 9 being “strong motion”. This rating procedure aimed to assess the effectiveness of the images in inducing an illusory motion effect. The ratings provided valuable information for subsequent analysis, as participants who did not perceive the illusory motion could be identified and potentially excluded from further analysis.

#### 2.1.1. Participants

We recruited 20 students (12 women, 8 men) from the University of Padova, with a mean age of 24.45, for voluntary participation. Participants had normal or corrected-to-normal visual acuity, no eye abnormalities, and were unaware of the purpose of the study. Informed consent was obtained from all participants and the study adhered to the principles of the Declaration of Helsinki. The experimental methods were approved by the Ethics Committee of the University of Padova (Prot. 3247).

#### 2.1.2. Apparatus

Individual testing took place in a quiet, dimly lit room, with participants sitting in front of a computer. The distance between the participant’s eyes and the monitor was measured at 57 cm and the participants were instructed to maintain this distance throughout the experiment. Visual stimuli were presented on a 21.5-inch Acer monitor with a refresh rate of 60 Hz. Stimulus generation was implemented using MATLAB PsychoToolbox-3.0.14 [40].

### 2.2. A Priori Power

Experiment 1 was designed with 10 repetitions for each level of the linear predictor and for each type of stimulus (illusory versus static images). The number of repetitions was decided according to guidelines that suggested at least 10 samples per linear predictor level and more than five subjects (or clusters) for each random effect [41,42]. To determine the sample size, a pilot study was initially conducted with 14 participants. Based on the parameter estimates from the pilot study, Monte Carlo simulations were performed using the SIMR package in R [43] with 1000 runs. Each simulation varied the number of simulated participants from 10 to 24. Power estimates were calculated based on the percentage of runs with an effect that exceeded the desired alpha of 0.05. The first sample size for which the power estimate exceeded 80% for the “time” variable (linear predictor relative to stimulus duration) was N = 20. With N = 20, the estimated power for the factor of interest “type” (type of stimuli: static vs. illusory) was above 95%, which ensured the detection of medium to small effects. For the “time by type” interaction, no specific prediction was made, so the experiment was not optimized to test it. With N = 20, an expected power of 50% was achieved for this interaction, allowing only detection of a large effect.

### 2.3. Data Analysis

Data analysis was performed in R [44]. The data were modeled using a generalized probit-linear mixed model for binomially distributed outcomes (GLMM). This approach can account for both within-subject and between-subject variability and has been found to be optimal for clustered categorical data in repeated measure studies [45,46]. We adopted a model-selection perspective as a complement to the more traditional hypothesis-testing approach [47]. Starting from a complete model that included all fixed terms of interest and their interactions, we derived candidate models by iteratively removing fixed terms and interaction terms. Only models that made sense based on the experimental design were considered. All candidate models had the same random structure and were fitted with Maximum Likelihood (ML) estimation using the “lme4” package in R [48]. Model comparison was performed using the small-sample-size corrected version of Akaike information criterion (AICc) [49] with the “dredge” function in the “MuMIn” package [50]. The optimal model was selected based on the smallest AICc value and the highest model weight, which represents the posterior model probability [51,52]. The full model had the number of “longer” responses as the dependent variable (referred to as “response” in the formula), stimulus duration as a within-subject continuous variable (“time”), stimulus category as a within-subject factor (“type”), and all their interactions. The random effects in the model accounted for the by-subject variation in the intercept and in the slope for the “time” variable. This random effect structure provided the best fit without compromising the model’s estimatability [53]. According to the lme4 package notation, the resulting full model can be summarized by the following formula:

(1) response∼time∗type+(time|subject)

After model selection, an omnibus test was conducted using Type-III Wald chi-square tests with the “Anova” function from the “CAR” package [54] over the final model, which was refitted with Restricted Maximum Likelihood (REML) estimation. Additionally, following the analytical approach in Moscatelli et al. (2012) [46], the Point of Subjective Equality (PSE) and the Just-Noticeable Difference (JND) for the two classes of stimuli were estimated from the GLMM using a bootstrap method with 500 samples. The PSE corresponds to the stimulus duration that leads to a 0.5 probability of a “longer” response and estimates the accuracy of the percept. In an ideal observer, the PSE would correspond to a duration of 2.0 s, and any deviation from this value indicates a bias in the percept. The JND, as an inverse function of the model slope, estimates the noise of the perceptual response. The median of the subjective rating given by the participants for each type of image was also reported as an evaluation of the perceived strength of the illusion.

### 2.4. Results

In Experiment 1, we compared the perceived duration between a static image and an illusory motion image. All subjects reported experiencing a sensation of motion with the illusory image, with a median motion strength rating of 6 (95% confidence interval [4, 7]). In contrast, the control image was perceived as completely still, with a median rating of 0 (95% confidence interval [0, 0]). Model selection revealed that the best model (AICc model 1 = 4145.012) included the main factors “time” and “type” but not their interaction. The best model was 2.5 times more likely (ratio between weights) than the model with the interaction (AICc model 2 = 4146.879). Importantly, the model without the factor “type” (AICc model 3 = 4152.417) was 41.5 times less likely than the selected best model, indicating that our experimental manipulation was effective and the factor “type” had high informative power. Detailed results from the model selection are presented in Table 1.

**Figure 2:**
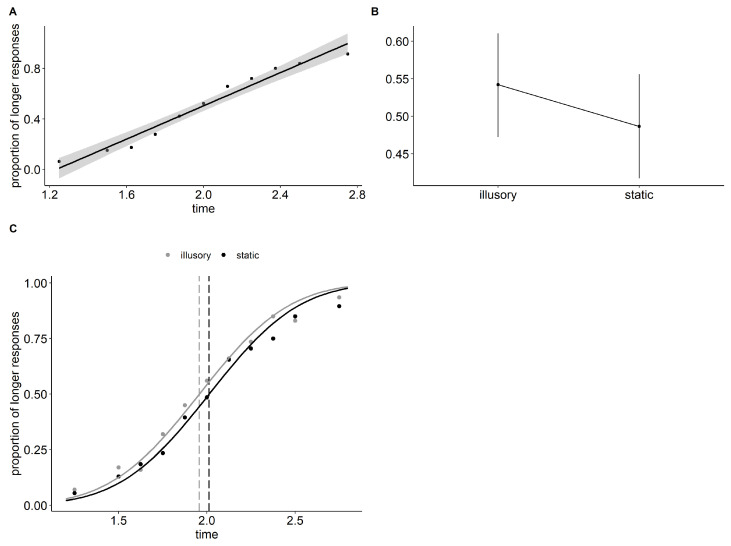
Time and type effects plots and estimated PSE for Experiment 1. (**A**) Proportions of “longer” responses as a function of time. Group average with linear regression and 95% confidence band. (**B**) GLMM estimated proportions of “longer” responses as a function of the type of stimulus (1 illusory vs. 2 static). Bars show 95% confidence intervals. (**C**) Psychometric functions and PSE (Point of Subjective Equality) for the illusory and static stimuli.

The omnibus Type-III Wald chi-square test revealed a significant main effect of “time” (df = 1, chisq = 115.878, *p* < 0.001) and a significant main effect of stimulus “type” (df = 1, chisq = 9.449, *p* = 0.002). The illusory stimuli received significantly more “longer” responses compared to the static stimuli (illusory − static = 0.139, std.err = 0.045, z = 3.074, *p* = 0.002). The psychometric fitting analysis showed that the Point of Subjective Equality (PSE) for the illusory stimuli was 1.958 s (C.I. = [1.888, 2.027]), while the PSE for the static stimuli was 2.013 s (C.I. = [1.943, 2.088]). The illusory stimulus had a 4% higher probability of being perceived as “longer” than the static stimulus, resulting in a PSE shift of 55 ms. The odd ratio for the illusory stimulus in this experiment was 1.07 (C.I. = [1.04, 1.14]). The absence of an interaction term in the final model constrained the slope to a common value for both illusory and static stimuli (JND = 0.269, C.I. = [0.229, 0.327]). Results are shown in Figure 2.

### 2.5. Discussion

In this experiment, we investigated the perceived duration difference between static and illusory motion images using a temporal discrimination task. Images with asymmetrical patterns induced an illusory perception of motion, rated by participants with a median score of 6 on a scale from 0 to 9. In contrast, images with symmetrical patterns were rated as static, with a median score of 0. Furthermore, we observed a 4% overall increase in “longer” responses for the illusory motion images compared to the static symmetric images. The consequent expansion in perceived duration was quantified by a PSE shift of 55 ms. Consistent with our expectations, this finding supports the hypothesis that illusory motion, similar to physical motion, expands the perceived duration of events. This perceived temporal expansion may potentially indicate the involvement of neural mechanisms that are shared between the processing of illusory motion and physical motion.

## 3. Experiment 2

Our ability to perceive the duration of an event is not governed by a dedicated organ but rather arises from the processing of temporal and non-temporal information gathered from our senses. This subjective experience of event duration is influenced by attention, memory, and perception, which are closely intertwined [55]. When judging whether a comparison stimulus (target) differs in duration from a previously presented reference stimulus (standard), judgments may also depend on the internal representation of the standard and the order of the targets [56,57,58]. Previous studies have demonstrated the relevance of individual memory access and updating abilities in temporal discrimination tasks [59]. Better updating and access capacity have been associated with more accurate and less variable performance [60]. In particular, the processing of stimuli lasting longer than one second activates the prefrontal cortex, as it necessitates the engagement of monitoring functions and the storage of temporal information in memory [61,62]. The ability to discriminate temporal intervals also depends on the variability of memory representations of standard duration and the prevention of contamination by subsequent stimuli throughout the task [56,63,64,65].

Based on these premises, one might expect that increasing the number of presentations of the reference duration at the beginning of the block or increasing its frequency between trials would enhance performance by improving the accuracy of the standard-duration representation in memory. However, contrary to this expectation, Jones and Wearden [66] demonstrated in 2003 that increasing the number of standard presentations from 1 to 5 had no significant effect on performance in a temporal generalization task. This result was later confirmed by Ogden and Jones in 2009 [67] for both visual and auditory modalities. Nonetheless, other studies have shown that memory for stimulus duration degrades over time and is susceptible to distortions [58,68,69]. In this second experiment, we aimed to investigate whether the previously observed time-dilation effect would persist despite reducing the frequency and overall number of standard-duration presentations throughout the experimental session. As a conceptual replication of the first experiment, we maintained the same number of trials (220) but reduced the overall number of presentations of the reference duration by increasing the number of trials per block from 10 to 55 (4 blocks instead of 22). Specifically, the reference duration was presented a total of 19 times, with 10 presentations at the beginning of the first block and 3 times at the beginning of each of the subsequent 3 blocks. Compared to Experiment 1, the total number of standard-duration repetitions was reduced from 73 to 19. Under these conditions, we anticipated a general decrease in performance due to increased uncertainty associated with the representation of the standard duration in memory. Additionally, by comparing the results of the two experiments, we aimed to examine whether the temporal expansion effect interacts with cognitive factors such as temporal memory.

### 3.1. Materials and Methods

Experiment 2 consisted of a total of 220 trials, which were divided into 4 blocks of 55 trials each. A total of 10 reference stimuli were presented at the beginning of the session, and 3 additional times at the beginning of each block. The discrimination task and instructions remained consistent with Experiment 1, including the procedure for collecting subjective ratings of illusion strength for each image category. The apparatus and analytical plan used in this experiment were identical to those employed in Experiment 1.

#### Participants

Twenty students (16 females and 4 males, mean age 23) volunteered to participate in the experiment. All participants had normal or corrected-to-normal vision, with no established visual pathology. They were naive to the purpose of the study, and informed consent was obtained from each participant before the study. The study adhered to the principles of the Declaration of Helsinki and received ethical approval from the Ethical Committee of the University of Padova (prot. 3247).

### 3.2. Results

Similar to the previous experiment, the median rating score for the perceived strength of illusory motion was 5 (95% C.I. = [3, 5]), while the control image received a median score of 0 (95% C.I. = [0, 0]). Once again, the best model (AICc = 3991.743) included “time” and “type” as main factors, but not their interaction. This model was 358.5 times more likely (based on the ratio of weights) than the model without the factor “type” (AICc model 3 = 4003.571). Removing the interaction improved the model fit by 2.5 times (AICc model 2 = 3993.619). Details of the model selection results are presented in Table 2.

**Figure 3:**
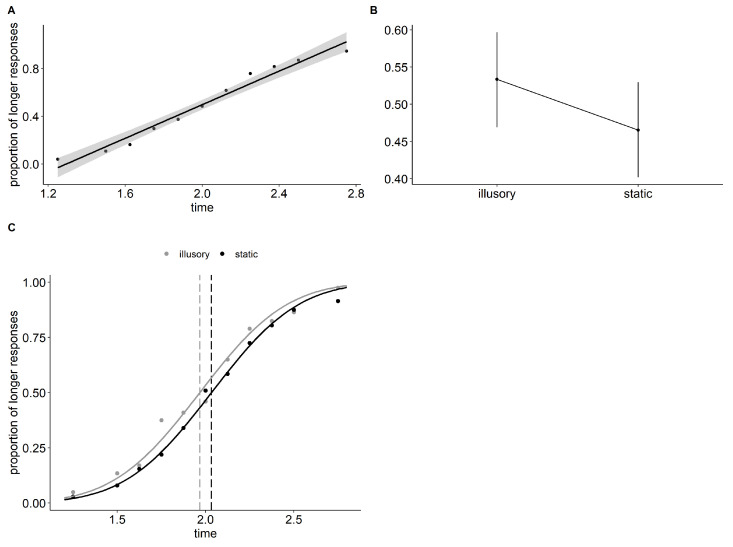
Time and type effects plots and estimated PSE for Experiment 2. (**A**) Proportions of “longer” responses as a function of time. Group average with linear regression and 95% confidence band. (**B**) GLMM estimated the proportions of “longer” responses as a function of the type of stimulus (1 illusory vs. 2 static) and texture. Bars show 95% confidence intervals. (**C**) Psychometric functions and PSE (Point of Subjective Equality) for the illusory and static stimuli.

The Type-III Wald chi-square test indicated a significant main effect “time” (df = 1, chisq = 354.779, *p* ≤ 0.001), and a significant main effect the “type” of stimulus (df = 1, chisq = 13.842, *p*≤ 0.001). Participants provided a higher number of “longer” answers in favor of the illusory stimuli compared to the static stimuli (illusory—static = 0.171, std. err = 0.046, z = 3.72, *p* < 0.001). The Point of Subjective Equality (PSE) for the illusory stimuli was 1.967s (C.I. = [1.905, 2.034]), while for the static stimuli, it was 2.034s (C.I. = [1.965, 2.102]). The probability of providing a “longer” answer increased by 4% for the illusory stimuli compared to the static stimuli, corresponding to a shift in PSE of 66 ms, as shown in Figure 3. The odd ratio for the illusory stimulus in this experiment was 1.09 (C.I. = [1.04, 1.14]). The Just-Noticeable Difference (JND) for both illusory and static stimuli was 0.260 (C.I. = [0.234, 0.288]).

### 3.3. Discussion

In Experiment 2, we manipulated the frequency and overall number of presentations of the standard duration throughout the experimental session. Similar to the previous experiment, the illusory motion images produced an expansion of perceived duration, resulting in a PSE shift of 66 ms compared to the static counterparts. We also replicated the small increase in the overall number of “longer” responses for the illusory images, which was 4% in the previous experiment.

Furthermore, despite a slightly lower median rating for the perceived strength of illusory motion in asymmetric images (Experiment 1 = 6; Experiment 2 = 5), the odds ratio for the illusory stimulus was slightly larger than in the previous experiment, and the estimated temporal expansion was 11 ms larger. Surprisingly, this difference was mostly due to a variation in the PSE of the static stimuli. There was an overall leftward shift in PSE with respect to Experiment 1 that was quantified in 0.009 ms for the illusory stimuli and 0.021 ms for the static stimuli. Instead, the difference in JND between Experiment 1 and 2 was 0.009. The striking similarity between the findings of the two experiments suggests that the perceptual mechanism underlying the temporal dilation effect is not influenced by the memory component required in the task.

## 4. Experiment 3

Velocity is defined as the ratio between the displacement made by a body and the time taken to complete it. Previous literature has shown a dependence between physical speed and the subjective temporal expansion of an event [9]. The specific relationship may depend on the configuration of the stimulus. For example, the perceived duration of an expansive concentric grating appears to obey temporal frequency or flicker rate [8], while for a linear grating, the perceived duration is reportedly affected by the speed of the stimulus [9]. The temporal frequency dependence aligns with early process accounts (V1), whereas the speed dependence requires more complex processing in higher-tier visual areas, such as area MT/V5 [70,71,72]. Complex influences of speed have been reported in more complex stimuli. For rotating blobs’, rotational frequency or angular velocity, rather than their local speed, predicted time dilation [73]. When a drifting plaid or a linear sum of two drifting sinusoids in different orientations is perceived as a coherent pattern motion, perceived duration dilates with increasing pattern speed, rather than component speed [11]. The way a moving diamond-shaped figure is perceptually organized consistently alters perceived speed and duration [13]. When environmental and retinal motions were dissociated through smooth pursuit eye movement, time dilation was more consistent with environmental motion [74].

Additionally, motion contrast increasing the subjective speed of a moving stimulus also increased subjective duration [75]. Furthermore, studies have reported complex influences of acceleration, deceleration, and other temporal changes in speed on perceived durations [12,76,77,78]. These findings imply that the perception of motion (as a result of complex motion processing) rather than its physical properties, plays a decisive role in motion-induced time dilation.

Experiment 3 aims to test the presence of a positive relationship between the perceived strength of illusory motion and its perceived duration. Previous research has shown that physical motion’s speed is related to the perceived duration, with higher speed resulting in longer perceived duration [9]. In this experiment, we expected a similar positive relationship between the “speed” of the illusion and the temporal expansion, indicating that stronger illusory motion leads to a larger perceived duration. RAP images similar to the one used in this study elicit an undulatory motion perception rather than a directional motion perception [36]. To quantify the “speed” of this type of motion, we incorporated in this study a subjective measure. In this third experiment, this was carried out not just to check the effectiveness of the experimental manipulation, but also to capture individual differences in the perception of the illusion and explore its association with the time-dilation effect.

### 4.1. Materials and Methods

The task and procedure in Experiment 3 were the same as those used in Experiments 1 and 2 except the stimulus duration levels were reduced from 11 to 9 by removing the longer and shorter duration. The total number of trials increased to 648, divided into 18 blocks consisting of 36 trials each. The session lasted for 1 h and 15 min (75 min). In Experiment 3, additional textures and a new reference stimulus were introduced (Figure 1). To create the second pair of textures, a 100° color subtraction was applied to the first texture. This green-orange pair, as described in the original work by Backus et al. (2005) [36], aimed to reduce the perception of illusory motion. The third pair was created by replacing all black regions of the green-orange pair with white, further reducing the illusory motion while maintaining stable pattern recognition under all conditions. The new reference stimulus consisted of a solid orange circle. In Figure 1 the pairs of textures have been ordered based on the expected illusion strength, with 1 representing the strong illusion, 2 representing the mild illusion, and 3 representing the weak illusion.

Each of the six textures was randomly presented 12 times per duration (12 repetitions × 9 duration levels). Ten reference stimuli were presented at the beginning of the session and three times at the beginning of each block. In this experiment, the green and orange references were randomly alternated. Both the reference and target stimuli were presented over a gray background. Based on the results obtained from Experiments 1 and 2, which did not show any significant effect of reference frequency, we selected a reference presentation frequency that was between the frequencies used in Experiments 1 and 2. The apparatus used in Experiment 3 was the same as that used in Experiments 1 and 2.

#### Participants

Twenty students (10 females and 10 males, mean age 23.45) voluntarily participated in the experiment. All participants had normal or corrected-to-normal vision and no established visual pathology. Participants were naive to the purpose of the study. Informed consent was obtained from all participants, and the study was conducted in accordance with the tenets of the Declaration of Helsinki. The experimental methods received approval from the Ethical Committee of the University of Padova (prot. 3247).

### 4.2. Data Analysis

The data analysis followed the same approach as highlighted in Experiments 1 and 2. In this case, the complete model included the continuous variable “time”, the categorical within-subject variable “type”, and the categorical variable “texture”, which referred to the type of stimulus with a strong illusion (texture 1), mild illusion (texture 2), or weak illusion (texture 3), along with the interactions between these variables. The random effects were the same as those in the previous two models, accounting for the subject-specific variation in the intercept and in the slope for the “time” variable. According to the notation of the lme4 package [48], the resulting full model can be summarized by the following formula:

(2) response∼time∗type∗texture+(time|subject)

Furthermore, to examine the effect of the perceived strength of the illusion on accuracy, we conducted a Type-III Wald chi-square test on a model that included accuracy as the dependent variable, time as the continuous variable, and subjective “rating” as the ordered variable. The random effects were the same as those in the previous models. To examine whether an eventual trend was linear or quadratic, orthogonal polynomial contrasts were performed among the levels of the “rating” variable.

### 4.3. Results

In this experiment, three pairs of images were used instead of one. As expected, the illusory image with the strongest supposed illusion was effective in producing a sensation of motion in all subjects. The median score for the rating of motion strength was 7 (95% C.I. = [6, 8]), and all subjects perceived motion while the control image was perceived as still (median = 0, 95% C.I. = [0, 1]). The mildly illusory image had a median rating of motion strength of 2.5 (95% C.I. = [1, 4]), and five of the subjects reported not perceiving the illusory motion at all. As expected, its control counterpart had a median value of 0 (95% C.I. = [0, 1]), but surprisingly, eight subjects attributed a score of 1. For the weak illusory image, the median rating of motion strength was 0.5 (95% C.I. = [0, 1]), with only half of the subjects reporting perceiving some sort of motion. On the other hand, its control image was always perceived as still (median = 0, 95% C.I. = [0, 0]). Model selection showed that the model including “texture”, “time”, and “type” as main factors, together with the “texture: type” interaction, provided the lowest value of AICc (14,210.27), indicating the “best” model. Results are displayed in Table 3.

The results of the omnibus test showed significant main effects for both “time” (df = 1, chisq = 166.883, *p* < 0.001) and “text” (df = 2, chisq = 8.982, *p* < 0.011). However, the “type” of stimulus did not yield a significant effect (df = 1, chisq = 2.623, *p* = 0.105). Additionally, there was a significant interaction between the “type” of stimulus and the “texture” (df = 2, chisq = 7.632, *p* = 0.022). Further Bonferroni corrected post-hoc comparisons revealed that within the illusory condition, the first texture was significantly different from the other two (texture 1 vs. 2: estimate = 0.147, std. err = 0.042, z = 3.472, *p* = 0.002; texture 1 vs. 3: estimate = 0.120, std. err = 0.042, z = 2.844, *p* = 0.013; texture 2 vs. 3: estimate = −0.027, std. err = 0.042, z = −0.633, *p* = 1). For the static condition, there was no significant difference between the three textures (texture 1 vs. 2: estimate = −0.018, std. err = 0.042, z = −0.416, *p* = 1; texture 1 vs. 3: estimate = 0.052, std. err = 0.042, z = 1.231, *p* = 0.655; texture 2 vs. 3: estimate = 0.07, std. err = 0.042, z = 1.652, *p* = 0.296) Similarly, the difference between illusory and static stimuli was significant for texture 1 (illusory − static = 0.117, std. err = 0.042, z = 2.763, *p* = 0.034), but not for texture 2 (illusory − static = −0.048, std. err = 0.042, z = −1.123, *p* = 1), or texture 3 (illusory − static = 0.049, std. err = 0.042, z = 1.161, *p* = 1).

**Figure 4:**
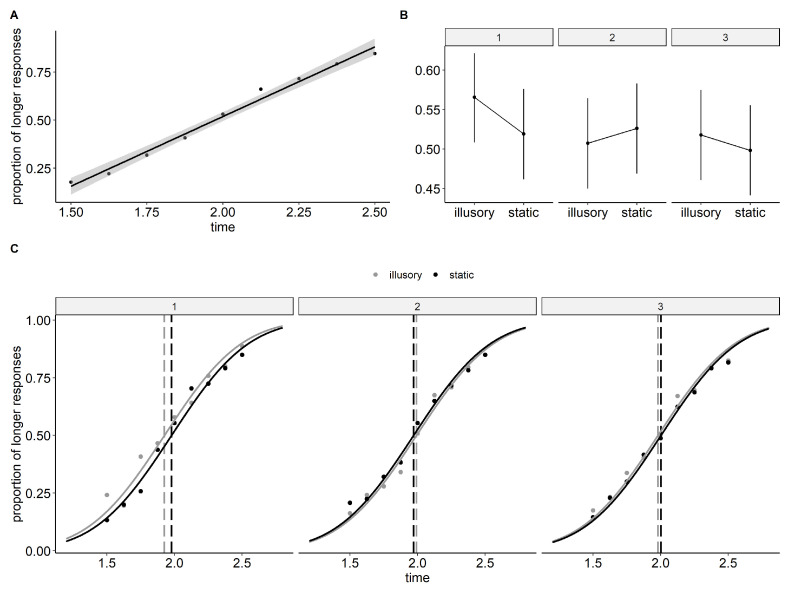
Time and type effects plots and estimated PSE for Experiment 3. (**A**) Proportions of “longer” responses as a function of time. Group average with linear regression and 95% confidence band. (**B**) GLMM estimated the proportions of “longer” responses as a function of the type of stimulus (1 illusory vs. 2 static) and texture. The pairs of textures have been ordered based on the expected strength of the illusion, with 1 representing the strong illusion, 2 representing the mild illusion, and 3 representing the weak illusion. Bars show 95% confidence intervals. (**C**) Psychometric functions and PSE (Point of Subjective Equality) for the three types of textures are divided into illusory and static stimuli.

The Point of Subjective Equality (PSE) for strong illusory stimuli was estimated to be 1.926 s (C.I. = [1.859, 1.986]), while the PSE for the static counterpart was 1.978 s (C.I. = [1.910, 2.036]). Mild illusory stimuli had a PSE of 1.992 s (C.I. = [1.923, 2.049]), and control images had a PSE of 1.971 s (C.I. = [1.9711.899, 2.031]). Finally, weak illusory stimuli had a PSE of 1.98 s (C.I. = [1.911, 2.037]), while control images had a PSE of 2.002 s (C.I. = [1.934, 2.056]). The Just-Noticeable Difference (JND) was the same for both illusory and static stimuli, with a value of 0.302 (C.I. = [0.261, 0.356]). Results are shown in Figure 4.

**Figure 5:**
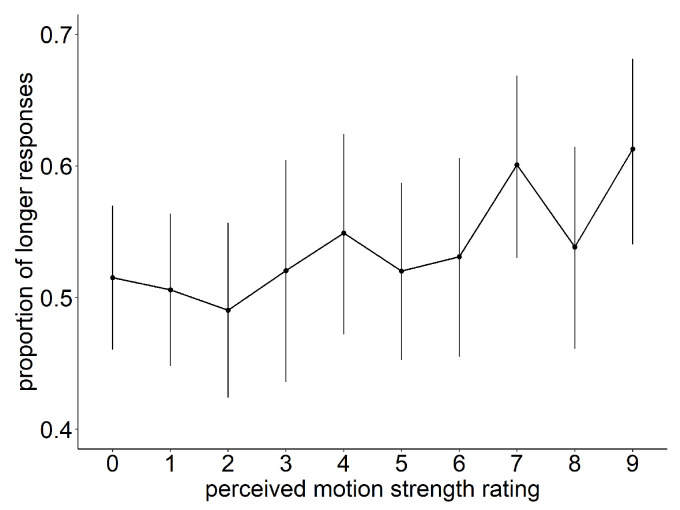
Perceived strength of illusory motion effect plot. The proportion of “longer” answers as a function of the subjective ratings of perceived motion strength. Bars show 95% confidence intervals.

To investigate the relationship between the proportion of “longer” responses and the perceived strength of the illusion, we analyzed the participants’ responses in relation to their rating of motion strength. The Type-III Wald chi-square test revealed significant main effects for both “time” (df = 1, chisq = 167.256, *p* < 0.001) and “rating” (df = 9, chisq = 30.107, *p* < 0.001). As shown in Figure 5, orthogonal polynomial contrasts indicate a significant positive linear trend between the strength of the illusory motion and the time-dilation effect (linear estimate = 0.232, std. err = 0.052, z = 4.457, *p* < 0.001).

### 4.4. Discussion

Experiment 3 aimed to investigate the relationship between perceived illusory motion strength and its perceived duration. We expected that manipulating the strength of illusory motion images would lead to different proportions of “longer” responses. Contrary to previous experiments, there was no significant main effect of stimulus “type” (static vs. illusory). The expected interaction between the stimulus “type” and “texture” was significant, but only texture 1, representing the strong illusion, was significantly different from the control texture. In this initial analysis, we could not verify whether decreasing the gradient of illusory motion elicited by the three textures also decreased the perceived temporal expansion, as textures 2 and 3 did not produce any time dilation. Surprisingly, texture 2 received a very low median rating of illusory motion, and some subjects reported not perceiving the illusory motion at all. Additionally, their control counterparts received similar ratings. Although the mild illusory image was sufficient to generate pupillary dilation compared to its control counterpart, as demonstrated by Beukema and colleagues (2017) [31], it may have been too weak to induce a time-dilation effect in our study. It should also be considered that individual susceptibility to the illusion varies, and participants can give different scores to the same image. Therefore, we tested this hypothesis with a different analysis, which no longer considered the subdivision between textures but directly considered subjective reports of illusion strength. Consequently, we conducted an analysis relating the responses to the participants’ ratings of motion strength. Finally, this analysis, in accordance with our hypothesis, demonstrated a linear trend between illusion strength and the time-dilation effect, suggesting that stronger illusions could produce a larger bias.

## 5. General Discussion

This study aimed to evaluate the time-dilation effect produced by illusory motion images compared to static stimuli and to investigate the relationship between perceived illusory motion strength and its perceived duration. Using RAP stimuli [31], we found evidence of a time-dilation effect generated by illusory motion images in three experiments. The average time-dilation effect for the texture with strong illusory motion was 58 ms across experiments. Although the magnitude of the shift in the PSE was small, the result proved to be robust. Textures 2 and 3 demonstrated a motion illusion too weak to provoke a sufficiently large temporal dilation that could be measured. However, in Experiment 3, we found a significant positive linear trend between the strength of the perceived illusion and the temporal dilation. These findings directly replicate previous findings with physical motion, although the temporal expansion induced by physical motion is greater (approximately 80 ms in biological motions [79]).

Cognitive factors, such as temporal memory, appear to have little or no influence on temporal expansion caused by illusory motion. This conclusion is supported by the remarkable similarity between the results of Experiments 2 and 3. Specifically, the reduction in the frequency and overall number of presentations of standard duration resulted in only a minimal variation in the precision of time estimation, as demonstrated by the very small difference in JND between Experiments 1 and 2 (JND difference = 0.009). Furthermore, the PSE shift caused by reference rarefaction was smaller for illusory stimuli (PSE difference = 0.009 ms) than for static stimuli (PSE difference = 0.021 ms).

Notably, in none of the three experiments did the interaction between “time” and “type” provide informative insights into model selection, and therefore, it was not included in the final model. This indicates that the JND was comparable between illusory and static stimuli, suggesting that the precision in performance was not influenced by the stimulus type.

Although the illusion of perceived motion partially depends on oculomotor events such as microsaccades [21,22], Au et al. found that the temporal dilation effect cannot be attributed to the motion of the object in retinotopic terms but rather spatiotopic terms [74]. Furthermore, within a virtual reality environment, Lo Verde et al. conducted a study that showed a time-dilation effect for apparent motion but not for retinal or world motion alone [80]. These findings challenge the notion that temporal expansion depends on retinal motion generated by ocular movements.

Previous studies have shown that saccades can affect perceived time, inducing both saccadic chronostasis [81,82] and saccadic time compression [19,83]. Although we did not actively monitor the eye movements of the participants, there was no inherent advantage for them in deviating from the instructions and shifting their gaze from the fixation point during stimulus presentation. Moreover, an eventual increase in involuntary eye movements caused by the illusory motion, at most, could have diminished—and not increased—the magnitude of the temporal expansion, given that smooth pursuit eye movements [84] and microsaccades [85] are found to cause temporal compression. Attention can also play a significant role in influencing time perception. For instance, research suggests that the perceived duration of an oddball stimulus tends to be overestimated in comparison to a non-odd stimulus [86]. When a moving stimulus is additionally more salient, it leads to an increase in temporal expansion due to the combined effects of attention and motion [86]. Although we cannot exclude that the illusory stimulus may be more salient than the static stimulus, a similar argument can be made for physical motion versus static motion. Notably, both illusory motion and physical motion can pre-attentively guide visual perception in a visual search, with illusory motion “popping out” in a manner analogous to physical motion, thus facilitating parallelized search [29].

Pupillary dilation can be considered an indirect measure of attention and action for external stimuli [87]. Beukema et al., using a similar set of stimuli, found comparable pupillary dilation between texture 1 and texture 2, but we identified a temporal dilation only for texture 1 [31]. Moreover, given the high number of trials, an eventual novelty effect of the illusory stimulus can be ruled out.

All things considered, the observed time-dilation effect is not contingent upon differential oculomotor or pupillary responses to illusory stimuli compared to static stimuli. Additionally, several other factors remain consistent between the illusory stimulus and the control stimulus, including total and mean luminance, total and mean contrast, element count, element arrangement, surface area occupied by individual elements, and overall stimulus area [31]. Therefore, aside from the perception of illusory motion, no other stimulus characteristics could have contributed to the observed temporal dilation.

As proposed by Kaneko et al. [9], the pacemaker/accumulator is specifically designed for visual perception and should be investigated within the realms of perceptual processing. We argue that the increase in the number of “longer” responses and the resulting shift in the PSE indicate a temporal dilation phenomenon similar to that achieved through physical motion. Therefore, the illusory motion stimulus can accelerate the pacemaker’s rhythm in a manner analogous to physical motion [9].

By comparing the results of Experiments 1 and 2, we found that the dilation in perceived duration is not influenced by the degradation of the comparison reference in temporal memory. This finding suggests that the effect of illusory motion on time perception is not affected by cognitive factors. This finding supports the idea that illusory motion, in addition to real motion, can be considered a fundamental visual feature [29].

Previous studies found that both illusory and physical motion activate similar neural networks. This network consists of several processing nodes ranging from V1a to MT+ and beyond [26,27,28]. Time perception is also governed by a distributed brain network, which partially overlaps with the processing of other continuous dimensions [88,89]. Early research proposed that the interaction between time and motion occurs primarily during the initial stages of visual cortical processing, particularly in the V1 area [8]. However, more recent studies have shown that higher-order visual processing, specifically in the middle temporal area (MT), also plays a significant role in motion-induced time dilation [90]. The finding that rTMS applied to V1 does not necessarily disrupt the subjective perception of illusory motion, suggests that the motion illusion may originate from MT+ which receives motion-related information directly, via retino-collicular rather than retino-thalamic pathways [91].

Studies based on non-invasive brain stimulation also show that area MT/V5 seems to process duration primarily for visual stimuli [62].

Hence, this intersection point between the processing of time, real motion, and illusory motion emerges as the prime candidate for the role of the visual pacemaker/accumulator postulated by Kaneko et al. [9].

We can hypothesize a connection between the increase in activity in MT/V5 and the perceived temporal expansion. The finding that temporal expansion increases with the strength of the illusion supports this hypothesis. In fact, both the illusory motion [91,92] and the real motion [75,93] seem to lead to higher activity in the middle temporal area (MT/V5). In summary, our findings support the hypothesis that perceived duration may be associated with the amount of neural activity evoked by the stimulus in the motion-sensitive middle temporal area (MT/V5) [94,95]. Future research could further explore this result by utilizing stronger illusory images and neurophysiological measures.

## 6. Conclusions

This study provides evidence that illusory motion generates a time-dilation effect, and the strength of the illusion influences this effect. The similarity of this effect to that produced by real visual motion suggests a shared neural substrate. The increased neural activity in this substrate is likely responsible for the time-dilation effect.

## Figures and Tables

**Table 1:** Model comparison table.

Model	Intercept	Type	Time	Type Time	df	AICc	Delta	Weight
1	−4.903	+	2.504	−	6	4145.012	0.000	0.705
2	−4.950	+	2.528	+	7	4146.879	1.867	0.277
3	−4.963	−	2.500	−	5	4152.417	7.405	0.017
4	−0.166	+	−	−	5	4181.130	36.118	0.000
5	−0.235	−	−	−	4	4188.446	43.433	0.000

+/− factor included or excluded.

**Table 2:** Model comparison table for Experiment 2.

Model	Intercept	Type	Time	Type Time	df	AICc	Delta	Weight
1	−5.096	+	2.589	−	6	3991.743	0.000	0.717
2	−5.048	+	2.566	+	7	3993.619	1.877	0.281
3	−5.162	−	2.580	−	5	4003.571	11.828	0.002
4	−0.174	+	−	−	5	4049.139	57.397	0.000
5	−0.251	−	−	−	4	4060.931	69.188	0.000

+/− factor included or excluded.

**Table 3:** Model comparison table for Experiment 3.

Model	Intercept	Texture	Time	Type	Texture Time	Texture Type	Time Type	Texture Time Type	df	AICc	Delta	Weight
1	− 4.160	+	2.236	+	−	+	−	−	10	14,210.27	0.000	0.364
2	−4.170	+	2.236	+	−	+	+	−	11	14,211.84	1.570	0.166
3	−4.180	+	2.237	+	+	+	−	−	12	14,212.26	1.999	0.134
4	−4.210	+	2.238	+	+	+	+	+	15	14,213.12	2.853	0.087
5	−4.180	+	2.237	+	+	+	+	−	13	14,213.83	3.568	0.061
6	−4.130	+	2.235	+	−	−	−	−	8	14,213.89	3.623	0.059

+/− factor included or excluded.

## Data Availability

The datasets for this study can be found in the https://osf.io/7cr8w/?view_only=5e1570aebe5f4742924d3c9b3cfd4257 (accessed on 7 September 2023) OSF repository.
